# Older Persons’ Use of Social Media for Communication During Hurricane Matthew

**DOI:** 10.1093/geroni/igaa057.2427

**Published:** 2020-12-16

**Authors:** Allison Gibson, Nancy Kusmaul, Ethan Engelhardt

**Affiliations:** 1 University of Kentucky, College of Social Work, Lexington, Kentucky, United States; 2 UMBC, Baltimore, Maryland, United States

## Abstract

Older persons are frequently identified as more vulnerable during natural disasters due to age-related changes and chronic conditions. Over the last decade, the use of social media has grown, even among the older adult population. While many communities and organizations have utilized social media as a platform to communicate news and information about natural disasters among the public, little is known regarding how older adults utilize social media to plan, evacuate, and recovery from natural disaster events. This study examined the experiences of 23 older adults (n=23) use of social media following Hurricane Matthew. Individuals were able to speak about what they perceived as helpful in the information communicated, and recommendations they had for how such communications could be improved. The presentation will conclude with recommendations on how individuals helping with evacuation and recovery can utilize social media as a communication resource for older adults during disasters. Part of a symposium sponsored by Disasters and Older Adults Interest Group.

